# Best precision in measuring Qp/Qs is achieved with quadratic, not linear, stationary tissue background correction in phase contrast velocity encoded CMR

**DOI:** 10.1186/1532-429X-18-S1-P245

**Published:** 2016-01-27

**Authors:** Jannike Nickander, Peder Sörensson, Einar Heiberg, Andreas Sigfridsson, Martin Ugander

**Affiliations:** 1 Karolinska Institutet, Stockholm, Sweden; 2 Department of Clinical Physiology, Lund University, Lund, Sweden

## Background

Cardiovascular magnetic resonance (CMR) flow quantification for determining the ratio of flow between the pulmonary artery and aorta (Qp/Qs) is used to quantify cardiac shunts. Post-processing methods, including linear or quadratic stationary tissue background correction have been proposed to improve accuracy and precision in flow quantification. However, it is not known which method improves the flow quantification the most, and if patient characteristics effect the correction. We evaluated changes in accuracy and precision of Qp/Qs following both linear and quadratic stationary tissue background correction, and investigated the effect on the precision of different patient characteristics. We hypothesized that the standard deviation of mean Qp/Qs would be reduced following stationary background correction compared to uncorrected measures.

## Methods

We enrolled consecutive patients (*n*=94, age 50 ± 16 years, 62% male) referred for clinical CMR at 1.5T (Siemens Aera) in whom the CMR report had no mention of cardiac shunts, malformations in the great vessels or persistent arrhythmias. A free-breathing non-segmented phase contrast velocity encoded sequence was used for through-plane velocity encoded imaging in the aorta and pulmonary artery. Flow quantification was performed both uncorrected and corrected for both linear and quadratic background phase correction using semi-automated methods with manual adjustments in freely available software (Segment version 2.0 R4534, Medviso AB, Lund, Sweden). Poor image quality was an exclusion criterion. Patient characteristics were dichotomized according to high and low values in relation to the median. Means were compared by the paired t-test (t), and SDs by the F test (F).

## Results

Patients with good image quality (n = 91, age 50 ± 16 years, 62% male) had mean Qp/Qs uncorrected 1.04 ± 0.110, Qp/Qs with linear correction 1.06 ± 0.090 (t p = 0.06, F p = 0.06), and quadratic correction 1.04 ± 0.087 (t p = 0.62, F p = 0.03). Patient characteristics where quadratic background correction decreased variability were male sex (p=0.049), low height (p=0.03), low cardiac output (p=0.04), a greater difference in stationary tissue area between anterior and posterior images halves for aortic and pulmonary images (p=0.04), and high angulation of pulmonary slice orientation in anterior-posterior (p=0.04) and right-left (p=0.04) directions.

## Conclusions

Quadratic, not linear, stationary tissue background correction improved the precision of Qp/Qs compared to uncorrected measures in a clinical population. Patient characteristics where precision was improved with background correction included male sex, low height, low cardiac output, relatively greater amount of anterior stationary tissue, and more angulated orientation of pulmonary artery images.

